# New Set of EST-STR Markers for Discrimination of Related *Papaver somniferum* L. Varieties

**DOI:** 10.3390/life14010072

**Published:** 2023-12-31

**Authors:** Šarlota Kaňuková, Katarína Ondreičková, Daniel Mihálik, Ján Kraic

**Affiliations:** 1 Department of Applied Biology and Genetics, Research Institute of Plant Production, National Agricultural and Food Centre, Bratislavska cesta 122, 92168 Piestany, Slovakia; sarlota.kanukova@ucm.sk (Š.K.); katarina.ondreickova@nppc.sk (K.O.); daniel.mihalik@nppc.sk (D.M.); 2 Department of Biotechnology, Faculty of Natural Sciences, University of Ss. Cyril and Methodius, Namestie J. Herdu 2, 91701 Trnava, Slovakia

**Keywords:** opium poppy, DNA typing, microsatellite markers, forensic analysis, genotype discrimination

## Abstract

*Papaver somniferum* L. is cultivated for its edible seeds and for the production of alkaloids. A serious problem in seed trade and processing is the intentional mixing of excellent food-quality seeds with non-food-grade-quality seeds. Tracking the correct or illegitimate handling of seeds requires an efficient method for discrimination and individualization of poppy varieties. As in human and animal forensics, DNA variable regions containing short tandem repeats (STRs) located either in non-coding DNA or in gene sequences (EST-STRs) are preferred markers for discrimination between genotypes. Primers designed for 10 poppy EST-STR loci not analyzed so far were tested for their discriminatory ability on a set of 23 related *P. somniferum* L. genotypes. Thirty-three EST-STR alleles were identified together. Their polymorphic information content (PIC) values were in the range of 0.175–0.649. The PI value varied in the range of 0.140–0.669, and the cumulative PI was 1.2 × 10^−5^. PI_sibs_ values varied between 0.436 and 0.820 and the cumulative value was lower (5.0 × 10^−3^). All analyzed genotypes were distinguished mutually, each with its own unique EST-STR profile. These newly developed EST-STR markers more effectively discriminated *P. somniferum* L. genotypes, even those genotypes whose DNA profiles were previously identical.

## 1. Introduction

Opium poppy (*Papaver somniferum* L.) is a plant species known for having two main legal utilizations. Primarily, poppy seeds are used for direct consumption and processing in households and the food industry. The seeds of the poppy varieties intended for consumption have a mild sweet and pleasant taste and aroma. In many countries, poppy seeds are an essential part of traditional dishes, and local and regional specialties are very popular with consumers. Opium alkaloids are a product of no less importance in poppy plant cultivation. These alkaloids are extractable from the dry, ripe poppy heads and terminal parts of the stems. The spectrum of alkaloids is diverse (morphine, codeine, thebaine, narcotine, papaverine) and variable in proportions and contents [1]. Special poppy varieties are bred for both ways of use. Varieties intended for the production of alkaloids, especially morphine, are bred and must also be grown according to certain rules. However, the quality of seeds from high-alkaloid varieties for consumption and food production is very low, even unusable. Nevertheless, they are sometimes mixed with high-quality seeds and thus used in the adulteration of seeds intended for food processing. Their mixtures with seeds of edible quality (blue color and sweet taste), after adding sugar or other substances, are sold, for example, in the form of poppy seed fillings to bakeries and the food industry. Food-grade poppy seeds intended for consumption and processing are also subject to other unfair practices. There are cases of unauthorized production and distribution of uncertified seeds. The motive for such action is the economic context related to the commercial exploitation of poppy seeds. Many problems also concern the breeding process itself or its finalization. A new registered variety should be legally protected. This also protects the rights of the author of the variety in the processes of certified seed production, plant cultivation, and any manipulation of the production of harvested seed. It is also important that the owner of a variety receives royalties for the use of his variety. Protection systems give rights to owners to control the production, distribution, and marketing of their seeds. The purpose is to provide financial compensation for the costs spent on breeding new varieties. A generally adopted protection system was established by the International Union for the Protection of New Varieties of Plants (UPOV). According to their rules, the protection of plant varieties through the UPOV convention is based on morphological parameters [2]. These characteristics are often not enough to ensure the protection of a given variety, which also applies to opium poppy varieties. However, there is potential for molecular techniques to provide new approaches to the processes of registration and legal protection of plant varieties. However, the use of DNA analysis techniques to solve such problems is still a matter of debate and awaits practical application.

Different DNA marker systems were tested for the differentiation of poppy varieties, including the RAPD, ISSR [3,4], and AFLP [5] markers. However, they have many disadvantages. Generally, the most applicable molecular markers within many plant species are microsatellites. Microsatellite markers are derived either from genomic DNA, referred to as short tandem repeat markers (STRs), or from cDNA, referred to as expressed sequence tags-short tandem repeats (EST-STRs). Both of these types of microsatellite markers have already been successfully used in the opium poppy [6,7,8,9]. However, distinguishing between varieties of the same species is very difficult when they are genetically closely related. This happens when newly registered varieties are created only from a limited number of alternating elite parents, if the availability of usable genetic resources is limited, or when their breeding is concentrated only on a small number of breeding workplaces. In this regard, poppy breeding is particularly specific, which is also confirmed by the limited number of varieties registered, subsequently introduced to the market, and cultivated in practice.

Considering the above, the aim of this study was to find and develop a new set of molecular markers derived from microsatellite sequences located in conservative (coding) sequences of the genomic *P. somniferum* L. DNA. The key parameter of such markers (EST-STR) should be the highest possible discriminating ability within the set of registered opium poppy varieties that are commercially used and cultivated in the most important growing areas.

## 2. Materials and Methods

### 2.1. Papaver somniferum L. Genotypes

The analyzed set included thirteen registered opium poppy varieties developed at the Research Institute of Plant Production, Malý Šariš, Slovakia (Albin, Bergam, Gerlach, Major, Maraton, Opal, Malsar), Oseva Pro, Ltd., Opava, Czech Republic (Orfeus, Orel, Racek, Redy, Sokol), and Saatbau Linz, Austria (Aristo). Some of these varieties have a dominant position in cultivation areas and seed production in Central Europe. However, information on the parental components used in the crosses of these varieties is essentially unpublished and unavailable. Two other breeding lines were developed at the Research Institute of Plant Production, Malý Šariš, Slovakia (MS 423, MS B2) and eight accessions (MSGZ-1, MSGZ-2, MSGZ-3, MSGZ-4, MS106, Hanácký modrý, GZ Afgánsky, UDS 01763) were obtained from the collection of *P. somniferum* L. genetic resources (Genebank of the Slovak Republic, Piešťany, Slovakia).

### 2.2. STR Analyses

Short tandem repeats were searched within the sequences of the *P. somniferum* L. genome, cultivar HN1, obtained using whole genome shotgun sequencing [10]. They are available in the GenBank^®^ database [11] (http://www.ncbi.nlm.nih.gov/genbank, accessed on 2 December 2019) in the genomic sequences of individual chromosomes (NC_039358.1–NC_039368.1). The SSRLocator software was used for this screening [12]. Primer pairs for amplification of EST-STRs were designed using the software BatchPrimer3 [13] (http://wheat.pw.usda.gov/demos/BatchPrimer3/, accessed on 2 December 2019). Their names (loci), nucleotide sequences, and repeat motives are in Table 1.

Genomic DNA was extracted from equivalent amounts of ninety to one hundred developing plants at stages 1–3 true leaves on main shoots using the Plant DNeasy Maxi kit (Qiagen N.V., Venlo, The Netherlands). Qualitative and quantitative parameters of isolated DNA were determined using agarose electrophoresis and spectrophotometry (using the NanoDrop 1000 Spectrophotometer, Thermo Scientific Inc., Waltham, MA, USA), respectively. PCR reactions were carried out in reaction mixtures containing 25 ng of template poppy DNA, 0.20 μM of each primer (Eurofins, Luxembourg City, Luxembourg), 0.2 mM dNTPs (Invitrogen, Carlsbad, CA, USA), 1 unit of recombinant Taq DNA polymerase (Invitrogen, Carlsbad, CA, USA), 1.5 μL of 10X Taq reaction buffer (final concentration of MgCl_2_ was 1.5 mM), and H_2_O to a total volume of 15 μL. PCRs were performed in the GeneAmp^®^ PCR System 9700 (Applied Biosystems, Foster City, CA, USA) using the following program: 3 min at 94 °C followed by 45 cycles of 45 s at 94 °C, 1 min at 54 °C, and 1 min at 72 °C. The final extension was 10 min at 72 °C.

Amplified EST-STRs were analyzed using electrophoresis in denatured polyacrylamide gels. An amount of 5 μL of PCR samples was mixed with 4 μL of loading buffer (4.8 g urea, 10 mL H_2_O, 0.05 g bromophenol blue, 10 mL, 10 mM NaOH), denatured at 100 °C for 2 min, and separated in 6% polyacrylacrylamide gel denatured by 7 M urea in 0.5X TBE buffer (1X TBE: 90 mM Tris-borate, 2 mM EDTA, pH 8.0). The parameters of separation in gels were a constant power of 40 W and a temperature of 50 °C. DNA in gels was stained with silver [14,15]. The size of STR alleles in base pairs (bp) was determined using Invitrogen^TM^ 10 bp DNA Ladder and Invitrogen^TM^ 20 bp DNA Ladder (Invitrogen, Carlsbad, CA, USA).

### 2.3. Genetic Analyses

The number of alleles (N_a_), number of effective alleles (N_e_), observed and expected heterozygosity (H_Obs_, H_Exp_), Shannon’s information index (I), probability of identity (PI), and probability of identity for siblings (PI_sibl_) were calculated using the software GenAIEx v. 6.5 [16]. The software Cervus v. 3.0 [17] was used to determine the polymorphic information content (PIC). The marker index (MI) and discrimination power (D) were calculated using the iMEC Online Marker Efficiency Calculator [18]. Simple regression analysis was performed using the statistical package Statgraphic 18^®^ Centurion (Statgraphics Technologies Inc., The Plains, VA, USA). Polymorphic STR markers were used in cluster analysis with the neighbor-joining method for grouping genotypes. Dendrograms were constructed using Jaccard’s similarity coefficient with the DARwin 5.0.158 statistical software [19].

## 3. Results

All ten primer pairs (Table 1), designed from the sequences of the *P. somniferum* L. genome (cultivar HN1) [10], were derived from the coding sequences of specific or uncharacterized proteins, respectively. The primer design was focused on EST-STRs containing trinucleotide tandem repeats. Allelic monomorphism was not detected in any of these analyzed loci, so all were included in the discrimination analyses. The sizes (lengths) of polymorphic alleles and the code of the original poppy genomic DNA sequence are presented in Table 1.

The genetic parameters of the discrimination system used based on EST-STRs are presented in Table 2.

There were 2–5 alleles identified at individual loci, and the length of amplified fragments containing variable EST-STRs was in the range of 127–232 bp. The total number of detected and evaluated alleles was 33, and the mean number of alleles per locus was 3.3. The number of effective alleles was lower than the number of alleles, and its mean value was 2.10. Heterozygosity was observed in six loci (PsTlFbP, PsPDRPRGA3, PsAcATE13lP, PsOT4l, PsUPLoc113286548ch6, PsHD2l); the other four were homozygous. The expected mean heterozygosity was 0.491. The observed mean heterozygosity was only 0.078, and, at 4 loci, it had a value of 0. Only in locus PsUPLoc113286548ch6, where 9 out of 23 poppy genotypes were heterozygous, the value was a little higher (0.391). Heterozygosity was identified mostly in breeding lines and accessions from the collection of genetic resources. This indicates a higher genetic heterogeneity in genotypes that did not go through the breeding process. On the contrary, varieties already registered showed high genetic homogeneity. They have gone through the process of increasing their uniformity, which is one of the requirements for new variety registration. The low mean value of H_Obs_ is an advantageous feature of this set of EST-STR markers from the point of view of practical application for forensic purposes. The results of discrimination analyses can then be interpreted more easily, thanks to the markers used for highly homozygous loci.

Shannon’s information index values for all individual loci as well as its mean value (0.827) were very low. Given the composition of the set of poppy genotypes, it was not surprising that it revealed very low genetic diversity and demonstrated their high genetic evenness.

An important parameter of marker system efficiency in genetic and forensic discrimination studies is the polymorphic information content (PIC). The higher it is, the greater its application value. The PIC values of the used markers were in the range of 0.175–0.649. The generally required PIC value is above 0.5, which 3 of the 10 tested markers had. Similar parameters to PIC are marker index (MI) and discrimination power (D). The MI shows the ability of primer pairs to detect polymorphic loci within genotypes, and D defines the ability of the typing method to distinguish between two randomly selected unrelated genotypes. Statistically significant (*p* ˂ 0.001) strong positive correlations were between PIC and MI (r = 0.938, R^2^ = 0.880) as well as between PIC and D (r = 0.935, R^2^ = 0.875).

**Figure 1:**
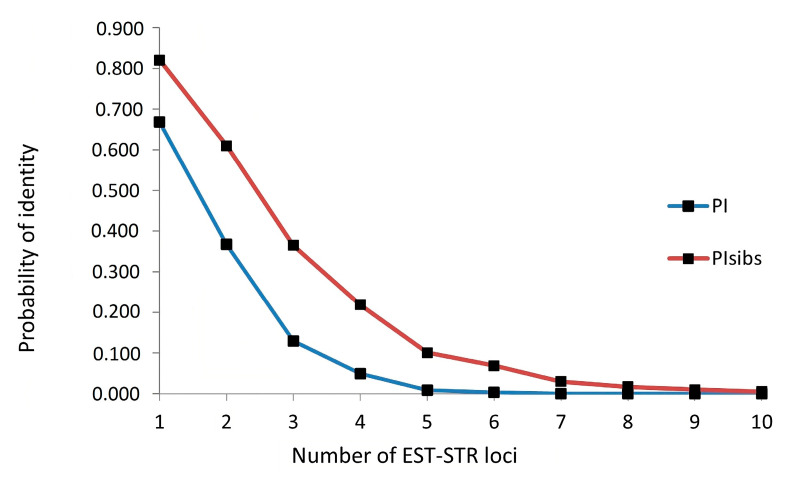
Cumulative probabilities of identity (PI, PI_sibs_) within *P. somniferum* L. genotypes estimated by used EST-STR markers.

The probability of identity (PI) varied between loci from 0.140 (locus PsOT4l) to 0.669 (PsTlFbP). PI values for individual loci showed how many genotypes matched at a given locus. For example, if only one was used, the most valuable locus (PsOT4I), only 14% of the genotypes would remain identical (Table 2). The analyzed set of opium poppies contained mainly registered varieties, especially Slovak and Czech. They were developed by breeders from non-random mattings of parents. These varieties certainly share significant parts of their genomes. They have a common history of breeding and originate from a common, limited genetic background of the parental components used in crosses. Therefore, PI_sibs_ values are perhaps even more important because they take these relationships into account. The obtained values of PI_sibs_ varied from 0.436 (locus PsOT4I) to 0.820 (PsTlFbP). Both PI and PI_sibs_ values logically decreased with increasing combinations of loci (Figure 1). 

However, from the point of view of the discrimination ability of the marker system, the most significant value is the cumulative probability of identity calculated from the probability of identity of all analyzed loci. The cumulative values of PI and PI_sibs_ were 1.2 × 10^−5^ and 5.0 × 10^−3^, respectively. Therefore, the simultaneous use of all 10 EST-STR markers gives a probability of identity between *P. somniferum* L. genotypes of 0.001% and 0.5%, respectively.

**Figure 2:**
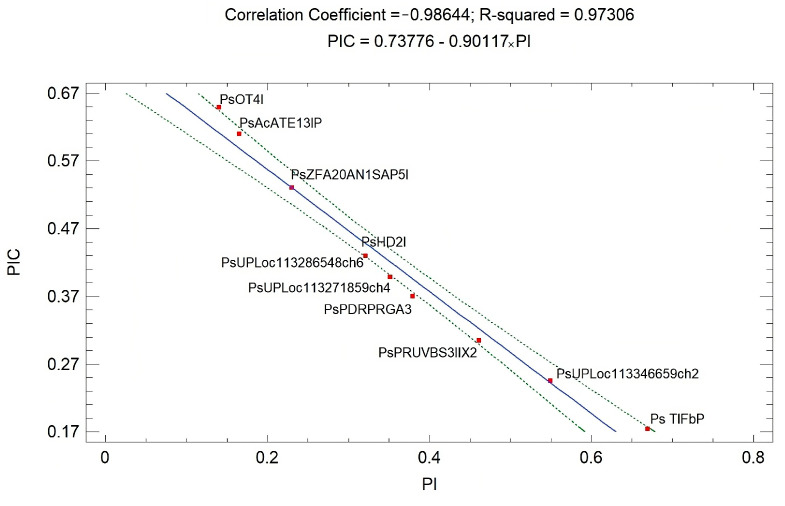
Positive correlation between PIC and PI values (10 points in the graph represent the 10 EST-STR markers used in the analyses).

A very strong linear negative correlation (*p* < 0.001) was found between the values of PIC and PI (r = −0.986). None of the markers were located outside the confidence limit (Figure 2). This confirms that the PIC value should be a decisive parameter for screening markers intended for genotype discrimination.

**Figure 3:**
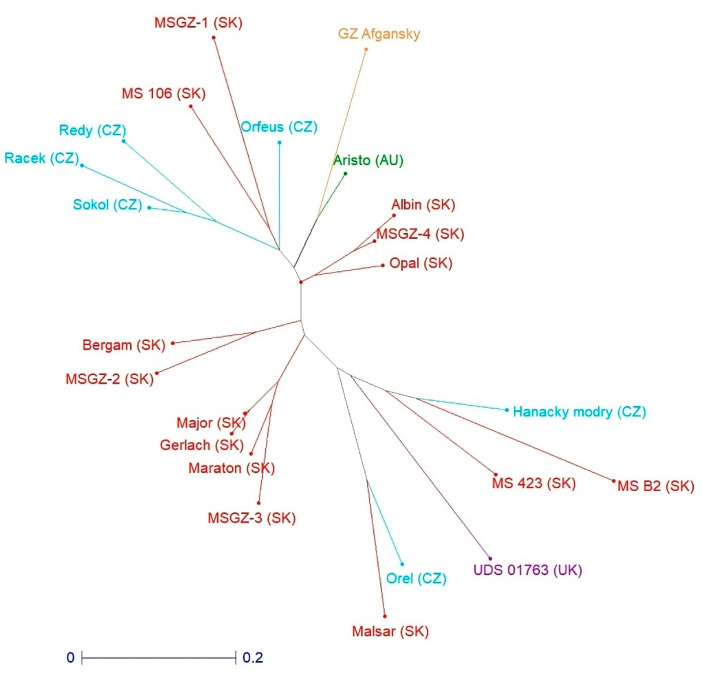
Discrimination between 23 analyzed *P. somniferum* L. genotypes with newly developed EST-STR markers.

Unequivocal identification of *P. somniferum* L. genotypes based on variations in EST-STR loci presents an output of the cluster analysis (Figure 3). All genotypes, especially registered varieties, were separated from each other, each with its own unique EST-STR identity.

A newly developed set of EST-STR markers presented in this study was more effective in *P. somniferum* L. discrimination than those developed previously [8]. It also differentiated the genotypes Malsar, Racek, and MS 423, which had identical DNA profiles at the EST-STR loci analyzed in that study.

## 4. Discussion

Several procedures based on DNA marker systems have already been developed for the discrimination of varieties in many crops, including opium poppy (*P. somniferum* L.). Some DNA markers were able to discriminate between *Papaver* species or *P. somniferum* L. subspecies. These have proven themselves, especially in the identification of narcotic and non-narcotic types of *Papaver* species. For this purpose, a Pscp1 variable number of tandem repeat (VNTR) markers derived from a variant region of the chloroplast genome [20] as well as SNP markers [21] were developed and validated. Different *Papaver* species were also distinguished using many SSR markers [22]. SSR markers were also used for ecological studies and analyses of the genetic diversity within *P. rhoeas* L. in agricultural ecosystems [23]. Other types of DNA markers used (AFLP, ISSR) were applied for the same purpose within *P. bracteatum* L. and several other *Papaver* species [24]. DNA (SNP) markers were also used for the analysis of intraspecific polymorphisms between biennial ornamental *P. nudicaule* L. genotypes [25]. During the search for the most suitable type of DNA marker for discrimination between *P. somniferum* L. varieties, almost all types were tested. Developmentally older types of DNA markers have no practical use in the analysis of discrimination within plant varieties in general, even in opium poppy, due to low differentiation competence and many technical and genetic disadvantages [3]. Either they are technically very demanding (RFLP), or show very low polymorphism and unreliability in reproducibility, making them unusable (RAPD, ISSR) [26]. The DNA barcode system based on the SNP markers derived from chloroplast DNA [27] also has limits for intraspecific discrimination within *P. somniferum* L. genotypes due to the high conservativeness of the chloroplast genome. The genotyping-by-sequencing analysis [28] approach is probably the most efficient but also the most technically, time-, and financially demanding. Functional applications were developed for human genetic forensics and some farm and wild animal species. However, forensic genetics currently represents a much wider range of applications, which increasingly include the analysis of non-human genetic material, including plant species. It provides supporting evidence in cases of identifying fraudulent activities in trading. Non-human forensic genetics is being developed by the increasing diversity of genetic markers and the introduction of faster, less error-prone, and cheaper methods and technologies [29]. They are unified in analytical approaches and standardized procedures and are based on STR typing systems. These efforts are leading to the fast and growing adoption of genotyping technology based on STR analyses [30,31]. However, even when using STR markers, the selection of highly polymorphic markers with good discrimination parameters is the most important. PIC and PI values are particularly crucial. Generally, markers with PIC > 0.5 are considered very informative, values between 0.25 and 0.50 are somewhat informative, and values lower than 0.25 are not very informative [32]. If such markers are not identified, then the discrimination power of analyses is low, and their use for individualization of genotypes is limited or even impossible. This has also been shown to be critical in the DNA discrimination of poppy genotypes. Low PIC values (below 0.300), even when using a larger number of SSR markers, did not lead to complete discrimination of opium poppy varieties [33]. This is the case if they are genetically related to each other because they were created by a limited number of parents with a similar breeding strategy and under similar environmental conditions. Even with SSR markers, if their selection is inappropriate, the differentiation of *P. somniferum* L. varieties may not lead to success [34]. Sometimes, even the selection and use of SSR markers with relatively high PIC values (0.284–0.767) could not distinguish the genotypes of *P. somniferum* L. [35].

The cumulative PI value using the set of EST-STR markers presented in our study was 1.2 × 10^−5^. It is a much better PI value for discriminating between opium poppy varieties than in our previous study (1.04 × 10^−3^) [9]. However, other EST-STR markers were used in this work; half of them were developed and published by other authors [6], and, together, they proved to be less effective. Even with PIC values, the lowest possible cumulative PI values should guarantee the good discrimination ability of markers. But, as with PIC values, this is not an absolute and universal indicator of the discrimination quality of markers in registered varieties that have gone through the breeding process. Even if the cumulative PI values are extremely low, a set of such markers may not be able to distinguish opium poppy varieties even within their small number [36].

Identifying and applying suitable EST-STR markers is somewhat more complicated compared to SSR/STR markers. These typically show lower polymorphism compared to STR markers due to their location in DNA coding regions. Therefore, a larger number of them should be screened to find highly polymorphic ones with the potential to discriminate between plant varieties. Sometimes, even a massive screening of EST-STR markers does not yield enough polymorphic markers to distinguish individual genotypes of *P. somniferum* L. This was confirmed by the screening of more than twenty thousand opium poppy-expressed sequence tags, from which only six polymorphic EST-SSR markers were developed [6]. Nevertheless, if highly polymorphic EST-STR markers are found, they are suitable discriminatory tools, mainly due to their genetic stability and high intra- and inter-laboratory reproducibility.

The majority of *P. somniferum* L. genotypes analyzed in this study (20 out of 23) were developed only in 2 places, one in Slovakia and the other in the Czech Republic. All of them are characterized by the production of seeds of high food-grade quality, and some of them also have an acceptable parallel production of alkaloids in poppy heads. Breeders in both locations have a common breeding history and use the same opium poppy germplasm in their breeding programs, where genetic diversity is limited by both number and genetic background. Genetically, very similar or identical parents are therefore found in the pedigrees of new varieties. Perhaps this is precisely why the pedigrees of registered poppy varieties are practically unavailable. High genetic relatedness is probably the most serious reason for the difficult discrimination between poppy varieties. This was also confirmed in previous studies, where some registered varieties could not be discriminated from each other [9,36,37]. In these studies, there was also a significant overlap between the analyzed varieties developed in Slovakia and the Czech Republic. In each of these studies, small groups of two to four of the analyzed varieties remained identical in their DNA profiles. Therefore, we continued to search, design, and test other EST-STR markers on the same set of *P. somniferum* L. varieties and genotypes, respectively.

The motivation in this study was to prepare a set with a low number of EST-STR markers, which, however, would be able to discriminate very effectively even very closely related registered varieties within the species *P. somniferum* L. Such markers are located at loci in which there are several allelic variants, the frequency of their occurrence is well-balanced, and they show the highest possible values of PIC and PI [38]. The EST-STR marker system presented in this study demonstrated considerable discriminatory potential for *P. somniferum* L. varieties. But this does not mean that it cannot be further improved. Many other suitable EST-STRs can be identified in the available nucleotide sequences of the *P. somniferum* L. genome. It is also possible to combine the presented markers with the best markers already found and published in other opium poppy studies (e.g., [37]). In this way, sets of primers for opium poppy forensic analyses could be created, which would already resemble the sets standardized for forensic studies in human and some animal species genomes.

## 5. Conclusions

A newly developed set of ten EST-STR markers presented in this study effectively characterized variation at these loci and uniquely discriminated genotypes within a selected set of *P. somniferum* L. varieties and genotypes. The analyzed opium poppy varieties originating from Slovakia and the Czech Republic have a very close origin, were developed using a similar breeding strategy, and have a related genetic background. They are difficult to distinguish from each other, as was presented in previous studies in which it was not possible to distinguish some of them from each other even with DNA markers. The need to distinguish them from each other, especially between registered varieties, has led to the development and testing of several types of DNA markers. The genetic parameters of the set of EST-STR markers developed and used in this study showed promising polymorphic information content (PIC) and probability of identity (PI) values, as well as the discriminatory potential of these markers. According to the presented results, it could be concluded that it is possible to create a relatively small set of EST-STR markers (~10) with excellent genetic parameters for unambiguous discrimination of genotypes, especially registered varieties of *P. somniferum* L., either classified as culinary, pharmaceutical, or with dual exploitation. If other suitable EST-STRs from other authors were added to the EST-STR markers developed in our study, a common set of markers could be established as a very efficient and universal DNA tool for validating and standardizing a DNA-based method for individualization of opium poppy genotypes. A better tool could be offered to investigate and control the various forms of illegal practices that can occur during the breeding process, the protection of authors’ rights, seed production, and trade with seeds before sowing and after harvest.

## Figures and Tables

**Table 1:** Characteristics of EST-STR loci, primer sequences, and allelic polymorphism.

Locus	Repeat Motif	Primer Sequence (5′-3′)	Polymorphic Alleles (bp)	GenBank^®^ Accession Number
PsTlFbp	(TGA)_6_	ACTTCACAATACCCATCCCAG	194, 197	XM_026526123.1
		CTTACACACACAAGCACAGGA		
PsUPLoc113346659ch2	(TGG)_10_	CATGGCCAGTACCGATGTTG	191, 194	XM_026590141.1
		GTAACCATCGGCGTTTAATGC		
PsUPLoc113271859ch4	(GAA)_12_	ACCCAATTGAGAATCCAGAAGA	223, 226, 232	XM_026521782.1
		CCACATCCTTACCTTCACATTCA		
PsUPLoc113286548ch6	(CTT)_10_	AGCCTGTACCTTATCAAACC	127, 133, 139	XM_026535133
		TTTTATGGTTTCCCGGATGA		
PsPDRPRGA3	(GAT)_6_	TTACAACTGCGCTGGGATTC	170, 190	XM_026523892
		ACACCGAAGTACTCATCATCCA		
PsAcATE13lP	(GA)_10_, (CTT)_14_	TCATTGGGAAAGCTTACCA	181, 183, 190, 200, 208	XM_026523258
		TCAAGTTCCATTCGTCTGT		
PsPRUVBS3lX2	(GAT)_8_	AGGGAGAAAGAAGAAGGAGT	226, 228	NC_039362.1
		TCTCCGATTTCTCTCCATCT		
PsOT4l	(GAA)_17_	AGTACCACACCAAGAAAACA	173, 179, 188, 191, 200	XM_026535173.1
		TCTAACTTCTTCAATCGGTG		
PsHD2l	(CTT)_10_	CCAACTAATGAAAACCCAGG	168, 171, 174, 176, 177	XM_026543538.1
		TCGATACATAAGAAGGCGAT		
PsZFA20AN1SAP5l	(T)_9_AAA(T)_14_, (GAA)_6_	CTGTCGTCTCTCTCAGTTAA	227, 228, 231, 232	XM_026571943.1
		TCAGATTTGAAATCCCCTCT		

**Table 2:** Genetic and discrimination parameters of EST-STR markers/loci.

Marker/Locus	N_a_	N_e_	H_Obs_	H_Exp_	I	PIC	MI	D	PI	PI_sibs_
PsTlFbP	2	1.24	0.130	0.198	0.344	0.175	0.194	0.194	0.669	0.820
PsUPLoc113346659ch2	2	1.41	0.000	0.294	0.462	0.246	0.287	0.300	0.549	0.744
PsUPLoc113271859ch4	3	1.92	0.000	0.491	0.777	0.399	0.480	0.502	0.351	0.598
PsPDRPRGA3	2	1.97	0.087	0.502	0.685	0.371	0.491	0.510	0.379	0.599
PsAcATE13lP	5	2.93	0.043	0.673	1.277	0.610	0.659	0.688	0.165	0.462
PsPRUVBS3lX2	2	1.60	0.000	0.385	0.562	0.305	0.510	0.534	0.461	0.678
PsOT4l	5	3.32	0.043	0.714	1.346	0.649	0.698	0.729	0.140	0.436
PsUPLoc113286548ch6	3	2.14	0.391	0.544	0.833	0.430	0.532	0.490	0.321	0.564
PsHD2l	5	1.90	0.087	0.483	0.916	0.430	0.472	0.492	0.321	0.594
PsZFA20AN1SAP5l	4	2.56	0.000	0.622	1.069	0.531	0.609	0.636	0.230	0.503
Mean	3.3	2.10	0.078	0.491	0.827	0.415	0.493	0.508	nc	nc

N_a_—number of alleles, N_e_—number of effective alleles, H_Obs_—observed heterozygosity, H_Exp_—expected heterozygosity, I—Shannon’s information index, PIC—polymorphic information content, MI—marker index, D—discrimination power, PI—probability of identity, PI_sibs_—probability of identity for siblings, nc (the average value has no informative value).

## Data Availability

Data generated and analyzed during this study are included in this article.
